# Emotional labor, workplace violence, and depressive symptoms in female Bank employees: a questionnaire survey using the K-ELS and K-WVS

**DOI:** 10.1186/s40557-018-0229-9

**Published:** 2018-03-12

**Authors:** Guang Hwi Kim, Hee Sung Lee, Sung Won Jung, Jae Gwang Lee, June Hee Lee, Kyung Jae Lee, Joo Ja Kim

**Affiliations:** Department of Occupational & Environmental Medicine, Soonchunhyang University Hospital, Seoul, South Korea

**Keywords:** K-ELS, K-WVS, Emotional labor, Workplace violence, Depression, Bank

## Abstract

**Background:**

In modern society, the scale of the service industry is continuously expanding, and the number of service workers is increasing. Correspondingly, physical and mental problems related to emotional labor are becoming a major social problem. In this study, we investigated the relationship between emotional labor, workplace violence, and depressive symptoms in female bank employees, which is a typical service industry.

**Methods:**

In this study, the Korean Emotional Labor Scale (K-ELS) and Korean Workplace Violence Scale (K-WVS) were distributed to 381 female workers in their 20s at a bank in Seoul, Korea. Data were obtained from 289 subjects (75.9%) and analyzed for 278 respondents, after excluding those with missing responses. We examined the relationship between emotional labor, workplace violence, and depressive symptoms, using multiple logistic regression analysis.

**Results:**

Among 278 subjects, 27 workers (9.7%) had depressive symptoms. “Emotional disharmony and hurt” (OR 2.93, 95% CI = 1.17–7.36) and “Organizational surveillance and monitoring” (OR 3.18, 95% CI = 1.29–7.86) showed a significant association with depressive symptoms. For workplace violence, the “Experience of psychological and sexual violence from supervisors and coworkers” (OR 4.07, 95% CI = 1.58–10.50) showed a significant association. When the number of high-risk emotional labor-related factors was 1 or more, 13.1% showed depressive symptoms. When the number of high-risk workplace violence-related factors was 1 or more, 14.4% had statistically significant depressive symptoms.

**Conclusions:**

A significant result was found for depressive symptoms related to Emotional disharmony, which is a sub-topic of emotional labor, and those at high risk for “Organizational surveillance and monitoring.” For workplace violence, depressive symptoms were high for the group at high risk for the “experience of psychological and sexual violence from supervisors and coworkers.” In this way, management of emotional disharmony, a sub-factor of emotional labor, is necessary, and improvements to traditional corporate culture that monitors emotional labor is necessary. Violence from colleagues and supervisors in the workplace must also be reduced.

IRB Approval No. SCHUH 2017–01-029. Registered 26 January 2017. Retrospectively registered.

**Electronic supplementary material:**

The online version of this article (10.1186/s40557-018-0229-9) contains supplementary material, which is available to authorized users.

## Background

The importance of the service industry has increased in modern society [1]. According to the National Statistical Office, as the scale of the service industry continues to expand in Korea, the role of service workers has also expanded. Correspondingly, the risk of service workers being exposed to verbal and physical violence from customers (e.g., being yelled at) is increasing at work, and physical and mental problems experienced by these employees are becoming a significant social problem [2].

Emotional labor refers to expressing only certain emotions with customers, or regulating emotions to meet job requirements [3]. Hochschild (1983) defined emotional labor as “the management of feelings to create a publicly observable facial and bodily display [4] .” To express required emotions in a job, it is necessary to adjust one’s emotions, which can cause stress due to incongruity [5]. Emotional labor can require the expression of greater emotion, involve strict rules, diversity, and emotional disharmony, and may result in low work satisfaction [6]. Emotional labor is also known to affect burnout [7] and depression [8].

Workplace violence can be related to emotional labor in the service industry. The Occupational Safety and Health Administration (OSHA) defines workplace violence as "any act or threat of physical violence, harassment, intimidation, or other threatening disruptive behavior that occurs at the work site." In addition, workplace violence, lack of social support, and an unpleasant work atmosphere are known to affect depressive symptoms [9]. To address these issues, workplace violence training workshops are offered to manage verbal, physical, and sexual violence experienced by employees in the workplace [10].

Many positions involve emotional labor such as those of airline workers, police personnel, nurses, etc. Studies have revealed that many professionals involved in emotional labor are prone to depression. Research also indicates that many occupations involving emotional labor affect the mental health of those performing such jobs [11–15]. Bank employees also perform emotional labor, as revealed in studies that show a relation between emotional labor and depressive symptoms [16]. Clearly, their job involves mental labor in dealing with customers, and most of them complain of mental suffering. The mental suffering of bankers is likely ignored when compared to that of other mental laborers who work in worse environments because bankers work in a relatively better environment. In addition, banking work environments may cause stress [17]; research shows verbal, mental, physical, and sexual violence experienced by bank employees to be related to mental health [18]. According to the National Statistical Office, the financial industry has declined due to continued deterioration of business performance, which may place a burden on workers in terms of structural adjustment, performance compulsion, and employment anxiety. This study aims to examine the relationship between workplace violence and depressive symptoms in female bank employees who work in a typical service industry that involves emotional labor.

## Methods

### Subjects

In total, 381 young female employees in commercial banks were selected to participate in this study. The survey was conducted in May 2015 using systematic questions asked during a health checkup. Of 289 respondents (response rate 75.9%), 278 were selected as the final research subjects, excluding 11 respondents whose responses were incomplete. We obtain ethical approval from the institutional review board (Approval No. SCHUH 2017–01-029) at Soonchunhayang university.

### Variables

The independent variables were sociodemographic factors, work-related factors, and levels of emotional labor and workplace violence (measured with the K-ELS and K-WVS, containing 24 questions each). Sociodemographic factors comprised age, marriage, education, regular exercise, smoking, drinking, and obesity. Work-related factors comprise occupation type, tenure, employment status, work hours per week, work hour sufficiency (First, we asked workers to work a few hours a week. Next, we asked them about the satisfaction level of the working hours. They had to answer in a yes/no format.), income, and income sufficiency. Age and working time cut - value were based on intermediate values. The dependent variable was level of depressive symptoms, measured with the Korean version of the Center for Epidemiologic Studies Depression Scale (CES-D). Scores of 21 and above on the CES-D indicated depression [19].

### Korean emotional labor scale (K-ELS) and Korean workplace violence scale (K-WVS) [20]

The K-ELS and K-WVS were developed in 2013 to evaluate emotional labor and workplace violence reflecting the Korean workplace and organizational culture; the validity and reliability of both scales have been confirmed. The K-ELS comprises “Emotional demands and regulation” (5 questions), “Overload and conflict in customer service” (3 questions), “Emotional disharmony and hurt” (6 questions), “Organizational surveillance and monitoring” (3 questions), and “Organizational support and protective systems” (7 questions). The K-WVS comprises “Experience of psychological and sexual violence from customers” (4 questions), “Experience of psychological and sexual violence from supervisors and coworkers” (4 questions), “Experience of physical violence from customers, supervisors, and coworkers” (2 questions), and “Organizational protective systems for workplace violence” (14 questions).

“Emotional demands and regulation”,“Overload and conflict in customer service”,“Emotional disharmony and hurt”,“Organizational surveillance and monitoring”, “Experience of psychological and sexual violence from customers”,“Experience of psychological and sexual violence from supervisors and coworkers”, “Experience of physical violence from customers, supervisors, and coworkers” are scored on the 4–3–2-1 Likert scale, “Organizational support and protective systems” and “Organizational protective systems for workplace violence” are scored on the 4–3–2-1 Likert scale and converted to 0 to 100 points I did it.

Questionnaire items reflecting specific and general aspects of organizational culture in Korea were derived and their reliability and validity were evaluated. Scores above a reference value for each gender were defined as high risk groups in a 2014 study. In this study, a cut-off value was presented based on the analysis result of the ROC curve (Receiver Operating Characteristic Curve) with the depressed and burnout collected from the questionnaire survey. Gender cut point was derived using all the fundamental elements of emotional labor and workplace violence converted as 0 to 100 points, and from the ROC analysis of the depressed and burnout, the area under the ROC curve is maximized based on the point of maximum value. ROC analysis was performed on the five factors of emotional labor and four factors of work violence in cases of depression and burnout. Based on this, the low cut point of depression and burnout was set as a reference value of the evaluation tool.

Evaluation of emotional labor reliability showed a satisfactory reliability coefficient of Cronbach’s alpha of 0.75 or more (0.761–0.904) for the following five factors. In addition, the Cronbach’s alpha value of all four factors in the lower part of the workplace violence showed a satisfactory reliability coefficient of 0.50 or more (0.509–0.968). [21]

### Data analysis

Chi-square tests were used to determine differences in the distribution of depressive symptoms according to sociodemographic and work-related factors. A multivariate analysis of K-ELS and K-WVS items and depressive symptoms was conducted. Then, after correcting for education, exercise, drinking, obesity, work hour sufficiency, and employment status, multiple logistic regression analyses were performed. Based on the data of this study, education that appears to be related to depression, inclusion of satisfaction with working hours, other known researchers obesity, known as a factor related to depressive symptoms, exercise, Drinking, employment status. The significance level was set at *p* < 0.05 and SPSS 14.0 was used for the analyses.

## Results

The mean age of the 278 female employees was 27.68 years (SD 2.64, range 20 to 30). The average score on the CES-D scale was 9.00, and the number of subjects with depressive symptoms was 27 (9.7%).

There was a statistically significant relationship between depressive symptoms and education level, and depressive symptoms and age; no other factors were significant (Table 1). The proportion of subjects aged 27 or younger with depressive symptoms was 18.2%; for those older than 27, it was 7.1%. In total, 7.6% of university graduates or higher, and 22.5% of college graduates or lower had depressive symptoms.

**Table 1:** Distribution of Depressive symptom according to Sociodemographic or Work-related factors

Variable		Normal	Depressive Symptom	
		N (%)	N (%)	*P*-value*
Age	27 or less	54 (81.8)	12 (18.2)	0.008†
	Above 27	197 (92.9)	15 (7.1)	
Marriage	Unmarried	214 (89.9)	24 (10.1)	0.61
	Married	37 (92.5)	3 (7.5)	
Education	University or above	220 (92.4)	18 (7.6)	0.003
	Community College or below	31 (77.5)	9 (22.5)	
Regular Exercise	Yes	162 (89.5)	19 (10.5)	0.546
	No	89 (91.8)	8 (8.2)	
Drinking	No	46 (93.9)	3 (6.1)	0.35
	Yes	205 (89.5)	24 (10.5)	
Obesity	Normal	246 (90.4)	26 (9.6)	0.561
	Overweight	5 (83.3)	1 (16.7)	
Job	Office work	76 (90.5)	8 (9.5)	0.935
	Loan counseling	53 (91.4)	5 (8.6)	
	Bank clerk	122 (89.7)	14 (10.3)	
Tenure(year)	Less than 3	111 (89.5)	13 (10.5)	0.697
	3 or above	140 (90.9)	14 (9.1)	
Employment Status	Regular worker	87 (92.6)	7 (7.4)	0.362
	Temporary work	184 (89.1)	20 (10.9)	
Work hour per week	51 or less	166 (92.7)	13 (7.3)	0.064
	Above 51	85 (85.9)	14 (14.1)	
Work hour sufficiency	Yes	178 (94.2)	11 (5.8)	< 0.001†
	No	73 (82.0)	16 (18.0)	
Income (won/10,000/year)	Less than 3000	37 (90.2)	4 (9.8)	0.861
	3000–3999	140 (89.2)	17 (10.8)	
	4000–4999	65 (92.9)	6 (7.1)	
	5000 or above	9 (90.0)	21 (10.1)	
Income sufficiency	Yes	172 (90.5)	18 (9.5)	0.844
	No	79 (89.8)	9 (10.2)	
	Total	251 (90.3)	27 (9.7)	

*by Chi-square test

†p < 0.05

Work hour sufficiency was statistically significantly related to depressive symptoms (Table 1). Depressive symptoms were present in 5.8% of those who were satisfied with their working hours, and in 18.0% of those who were not satisfied.

With respect to emotional labor, depressive symptoms were present in 6.3% of those at low risk for “emotional disharmony and hurt” and 20.0% of those at high risk. Furthermore, depressive symptoms were found in 6.6% of the group at low risk for “experience of psychological and sexual violence from supervisors and coworkers” and 23.5% of the high-risk group. Other factors related to emotional labor and workshop violence did not show statistically significant differences (Table 2).

**Table 2:** Differences in Depressive symptom by Emotional labor-related factors and Workplace violence-related factors

Variable	Normal Depressive Symptom		
	N (%)	N (%)	*P*-value*
Emotional labor			
Emotional demanding and regulation			
Low risk group	178 (92.2)	15 (7.8)	0.1
High risk group	73 (85.9)	12 (14.1)	
Overload and conflict in customer service			
Low risk group	191 (92.3)	16 (7.7)	0.057
High risk group	60 (84.5)	11 (15.5)	
Emotional disharmony and hurt			
Low risk group	195 (93.8)	13 (6.3)	< 0.001†
High risk group	56 (56.0)	14 (20.0)	
Organizational surveillance and monitoring			
Low risk group	192 (93.7)	13 (6.3)	< 0.001†
High risk group	59 (80.8)	14 (19.2)	
Organizational supportive and protective system			
Low risk group	180 (91.8)	16 (8.2)	0.178
High risk group	71 (86.6)	11 (13.4)	
Workplace violence			
Experience of psychological and sexual violence from customer			
Low risk group	138 (93.2)	10 (6.8)	0.076
High risk group	113 (86.9)	17 (13.1)	
Experience of psychological and sexual violence from supervisor and coworker			
Low risk group	212 (93.4)	15 (6.6)	< 0.001†
High risk group	39 (76.5)	12 (23.5)	
Experience of physical violence from customer, supervisor and coworker			
Low risk group	245 (90.1)	27 (9.9)	0.417
High risk group	6 (100.0)	0 (0)	
Organizational protective system for workplace violence			
Low risk group	239 (90.9)	24 (9.1)	0.167
High risk group	12 (80.0)	3 (20.0)	
Total	251 (90.3)	27 (9.7)	

*by Chi-square test

†p < 0.05

After performing the variable analysis only to see the relationship between emotional labor and workplace violence under the depression in relation to workplace factors, multiple logistic regression analyses were performed, correcting for individual characteristics. “Emotional disharmony and hurt” (OR 2.93, 95% CI = 1.17–7.36) and “Organizational surveillance and monitoring” (OR 3.18, 95% CI = 1.29–7.86) showed a significant association with emotional labor. For workplace violence, “Experience of psychological and sexual violence from supervisors and coworkers” (OR 4.07, 95% CI = 1.58–10.50) showed a significant association (Table 3).

**Table 3:** Odds ratios for Depressive symptom according to emotional labor and Workplace violence

Variable		Crude OR (95% CI)	Adjusted OR* (95% CI)
Emotional labor			
Emotional demanding and regulation (ref. Low risk group)	High risk group	1.95 (0.87–4.37)	1.43 (0.58–3.48)
Overload and conflict in customer service (ref. Low risk group)	High risk group	2.19 (0.96–4.97)	1.61 (0.63–4.08)
Emotional disharmony and hurt (ref. Low risk group)	High risk group	3.75 (1.67–8.44)†	2.93 (1.17–7.36)†
Organizational surveillance and monitoring (ref. Low risk group)	High risk group	3.51 (1.56–7.87)†	3.18 (1.29–7.86)†
Organizational supportive and protective system (ref. Low risk group)	High risk group	1.74 (0.77–3.94)	1.43 (0.59–3.43)
Workplace violence			
Experience of psychological and sexual violence from customer (ref. Low risk group)	High risk group	2.08 (0.92–4.71)	1.60 (0.67–3.85)
Experience of psychological and sexual violence from supervisor and coworker (ref. Low risk group)	High risk group	4.35 (1.89–10.00)†	4.07 (1.58–10.50)†
Experience of physical violence from customer, supervisor and coworker (ref. Low risk group)	High risk group	0 (0)	0 (0)
Organizational protective system for workplace violence (ref. Low risk group)	High risk group	2.49 (0.66–9.44)	1.66 (0.40–6.87)

*Adusted for Age, Obesity, Education, Exercise, Drinking, Work hour sufficiency, Employment Status

†p < 0.05

When the number of high-risk emotional labor-related factors was 1 or more, 13.1% showed depressive symptoms. When the number of high-risk workplace violence-related factors was 1 or more, 14.4% had statistically significant depressive symptoms (Table 4).

**Table 4:** Distribution of Depressive symptom according to number of High risk of Emotional labor-related factors and Workplace violence-related factors

Variable		Normal	Depression Symptom	
		N (%)	N (%)	*P*-value*
Number of High risk of Emotional Labor	None	105 (95.5%)	5 (4.5%)	0.019†
	1 or above	146 (86.9%)	22 (13.1%)	
Number of High risk of Workplace violence	None	120 (96.0%)	5 (4.0%)	0.004†
	1 or above	131 (85.6%)	22 (14.4%)	
	Total	251 (90.3)	27 (9.7)	

*by Chi-square test

†p < 0.05

Next, we performed multiple logistic regression analyses, correcting for individual characteristics. We found a significant association between having 1 or more high-risk workplace violence-related factors and risk for depression (OR 3.14, 95% CI = 1.11–8.86) (Table 5).

**Table 5:** Odds ratios for Depressive symptom according to number of high risk of Emotional labor-related factors and Workplace violence-related factors

Variable		Crude OR (95% CI)	Adjusted OR* (95% CI)
Number of High risk of Emotional Labor	None		
	1 or above	3.16 (1.16–8.63)†	2.59 (0.90–7.47)†
Number of High risk of Workplace violence	None		
	1 or above	4.03 (1.48–10.98)†	3.14 (1.11–8.86)†

*Adusted for Age, Obesity, Education, Exercise, Drinking, Work hour sufficiency, Employment Status

†p < 0.05

## Discussion

This study investigated the relation between emotional labor and depressive symptoms using the K-ELS and K-WVS, and CES-D. Overall, 9.7% of female bank workers in this study experienced depressive symptoms, lower than the proportions of 18.6% of female workers in all industries and 14.8% of those working in financial institutions and insurance-related industries [16]. This may have been because workers who participated in the questionnaire were unable to understand their own symptoms, and because all the workers included in the study were from a single company. Some differences in the risk factors could also affect depressive symptoms. Because the questionnaire survey was conducted at the same time as the health checkup, there was an additional effect regarding concern about the disadvantage that could result in the personnel evaluation based on the result of the medical examination.

“Emotional disharmony and hurt” and “organizational surveillance and monitoring” were related to three times higher depressive symptoms in the high-risk group. Emotional disharmony is known to have a negative emotional influence, such as feelings of depression, because of the existence of “a discrepancy between what is expected and what is experienced [3] .” In concordance, this study showed that “emotional disharmony and hurt” had an effect on depressive symptoms.

“Organizational surveillance and monitoring” is considered to be related to the workplace environment. Organized monitoring of emotional labor with customers results in stricter emotional labor requirements for workers [22]. This accelerates the emotional labor-emotional disharmony-emotional exhaustion sequence, which also affects depressive symptoms [23]. Therefore, emotional disharmony should be targeted first, and organizations that monitor emotional labor should be aware of the consequences.

Those at high risk for the “experience of psychological and sexual violence from supervisors and coworkers” had a tendency of four times higher depressive symptoms. This factor expresses the level of exposure to mental and sexual violence and experience level from colleagues and supervisors in the workplace, indicating that the high-risk group has a relatively high percentage of depression. This is similar to existing research results on violence related to work increasing the risk of stress disorders and depression [24]. Thus, violence in the workplace has a greater influence on employees than on customers. Indeed, violence from colleagues and superiors at work has an ongoing impact unless the workplace or office is changed. In addition, workplace violence may even affect those who do not work with customers, such as general clerks and production workers; therefore, research on different types of occupations is necessary.

A relationship between depression and high risk groups using the K-ELS and K-WVS was shown. Emotional labor before correction and all violent items in the workplace After the correction, it became statistically significant by violence in the workplace. This shows that questionnaires can be used for high-risk groups management. If we can conduct research on whether there are differences in depressive symptoms depending on the presence or absence of high risk or the number of them for multiple occupations through additional research in the future, programs to manage high-risk individuals in terms of dimension can be introduced.

Several existing studies have shown a relationship between emotional labor, workplace violence and depression, and low work satisfaction, and characteristics of emotional labor and workplace violence have been presented [25]. Because of the characteristics of emotional labor and violence in the workplace, evaluation is primarily conducted through questionnaires; however, since no standardized questionnaire existed in Korea, we translated a questionnaire being used overseas. Based on the need for standardized Korean evaluation tools, K-ELS and K-WVS were announced in 2013 [20], after which applied research was conducted [21].

This study had several advantages in studying the relationship between emotional labor, workplace violence, and depression.

First, we used a questionnaire reflecting social and cultural aspects of Korea. In past research, foreign questionnaires were translated and used; therefore, social and cultural aspects of Korea were not reflected, and there was a limit to the questionnaire differences between studies. Thus, results of the questionnaire used in this study are more objective and match the characteristics of Korea. In addition, comparative analysis and integrated research with the research star group or business establishment will be easier to conduct in the future.

The second advantage of this study is that we simultaneously investigated emotional labor and workplace violence. Not many previous studies have simultaneously investigated these variables. This allowed for a more comprehensive understanding of the causes of depressive symptoms in workers.

Third, this study targeted a single company environment. The work environment, such as the atmosphere at the workplace, the welfare of the staff, rules to be protected, work intensity, working hours, etc., can be different for each company. Emotional labor and violence in the workplace are affected by the work environment. Even in the same industry, there can be differences in the working environment depending on the place of business or company. In this study, because we focused on employees working in the same workplace environment and corporate culture, a more objective approach was possible.

The limitations of this study are as follows. First, the sample was small. However, it is meaningful to conduct research in one environment, at a single office. Second, the age of the study subjects was limited to 30 years old or younger. This is because we targeted workers who had not been working long. However, workers who deal with many customers and are exposed to emotional labor typically have a shorter length of service. Therefore, it is meaningful to target samples that face more emotional labor. Third, banking is an occupation that involves emotional labor, but banks do not have representation. Several other workers are involved in emotional labor work in an even worse environment, such as those in the security divisions, market workers, other service industries, etc. Banks offer a good working environment, such as a relatively high annual salary and educational background, and hence, they are not a priority research subject. However, there are examples of bank workers who commit suicide due to depression arising out of mental suffering, such as pressure on performance; these are also subjects that society needs to protect, and therefore, research on them should progress. Fourth, we wanted to see if the number of items belonging to high risk increased the probability of going through a depression phase; but in this study, it was found that respondents showed high risk of becoming depressed even with just two or more items. Therefore, we were unable to ascertain the reason for this, but further research with more data may help us understand the reason for this (see Tables 4 and 5). Finally, we only targeted women, and therefore could not examine gender differences. However, according to a report of financial talent in 2014, including a forecast of supply and demand, announced by the Financial Committee, the proportion of women working in the financial area is highest in sales and marketing departments. It says that it is judged as reflecting the practices of the financial community utilizing female human resources to contact customers. Thus, the proportion of women is high in positions that are likely to involve emotional labor immediately after contacting customers. In addition, comparative studies by country show a tendency for the prevalence of depression to be higher in women than in men in all countries [26] (Additional file 1). Therefore, research such as this, on women who have a high possibility of exposure to emotional labor and have relatively high sensitivity to depressive symptoms is necessary.

## Conclusion

This study investigated the relationship between emotional labor, workplace violence, and depressive symptoms, using the K-ELS and K-WVS. A significant result was found for depressive symptoms related to Emotional disharmony, which is a sub-topic of emotional labor, and those at high risk for “Organizational surveillance and monitoring.” For workplace violence, depressive symptoms were high for the group at high risk for the “Experience of psychological and sexual violence from supervisors and coworkers.” In addition, those in the high-risk group for one or more factors had higher depressive symptoms compared to those at high risk for none.

It is very important to examine the social causes that could lead to depressive symptoms. If social and working environments that lead to depressive symptoms can be improved, this will help to prevent depression. In this way, management of emotional disharmony, a sub-factor of emotional labor, is necessary, and improvements to traditional corporate culture that monitors emotional labor is necessary. Violence from colleagues and supervisors in the workplace must also be reduced.

## Additional file

Additional file 1: Korean Emotional Labor Scale and Korean Workplace Violence Scale ( K-ELS & K-WVS). (DOCX 19 kb)

## Abbreviations

CI: Confidence Interval
K-ELS: Korean Emotional Labor Scale
KRW: Korean Won
K-WVS: Korean Workplace Violence
OR: Odds Ratio
