# Supplementary material for: Emotional labor, workplace violence, and depressive symptoms in female Bank employees: a questionnaire survey using the K-ELS and K-WVS
Source: Ann Occup Environ Med. 2018 Mar 12;30:17. doi: 10.1186/s40557-018-0229-9 (PMC5848577; doi:10.1186/s40557-018-0229-9)
Supplement: Supplementary file 1 — Korean Emotional Labor Scale and Korean Workplace Violence Scale ( K-ELS & K-WVS). (DOCX 19 kb) [file 40557_2018_229_MOESM1_ESM.docx]

Korean Emotional Labor Scale and Korean Workplace Violence Scale ( K-ELS & K-WVS )

| The next questionnaire was created to evaluate the level of your emotional labor. Please respond to the following questionnaire based on the current work performance situation by selecting the option that most closely reflects your thoughts. |
| --- |

| Question | Strongly disagree | Disgree | Agree | Strongly agree |
| --- | --- | --- | --- | --- |
| 1. I continually strive to not express negative emotions to customers | 1 | 2 | 3 | 4 |
| 2. While interacting with customers, I have to express my emotions as requested by the company | 1 | 2 | 3 | 4 |
| 3. I hide my feelings while interacting with business customers | 1 | 2 | 3 | 4 |
| 4. In order to carry out routine tasks, efforts to adjust  emotions are necessary | 1 | 2 | 3 | 4 |
| 5. Emotions expressed while interacting with customers differ from actual emotions | 1 | 2 | 3 | 4 |
| 6. I have to deal with aggressive customers | 1 | 2 | 3 | 4 |
| 7. I have to deal with customers who need my ability and authority | 1 | 2 | 3 | 4 |
| 8. There are difficulties in conducting business based on unreasonable requests of customers | 1 | 2 | 3 | 4 |
| 9. My pride is hurt when responding to customers | 1 | 2 | 3 | 4 |
| 10. I feel embarrassed when I do not express my feelings to customers | 1 | 2 | 3 | 4 |
| 11. My emotions are felt like products when responding to customers | 1 | 2 | 3 | 4 |
| 12. Embarrassing emotions remain when I answer customers  even after work | 1 | 2 | 3 | 4 |
| 13. I get scratches of my heart in the process of contacting customers | 1 | 2 | 3 | 4 |
| 14. Even if I am physically tired, I must do my best for my customers, so I feel emotionally exhausted | 1 | 2 | 3 | 4 |
| 15. As per the workplace demands, it is possible to monitor whether I am dealing well with customers (through CCTV) | 1 | 2 | 3 | 4 |
| 16. Customers’ evaluation affects performance and personnel evaluation | 1 | 2 | 3 | 4 |
| 17. When problems occur during customer service, I am unfairly treated at the workplace, although it is not my fault | 1 | 2 | 3 | 4 |
| 18. When problems occur during customer service, appropriate measures are taken at the workplace | 4 | 3 | 2 | 1 |
| 19. There are formal systems and procedures in the workplace that solve and support problems that occur during the customer service process | 4 | 3 | 2 | 1 |
| 20. The workplace will receive the bruises of my heart that I received in the process of customer service | 4 | 3 | 2 | 1 |
| 21. My boss helps solve the problems that occur during customer service | 4 | 3 | 2 | 1 |
| 22. Colleagues can help solve the problems that occur during customer interactions | 4 | 3 | 2 | 1 |
| 23. There are established action guidelines and manuals concerning customer service in the workplace | 4 | 3 | 2 | 1 |
| 24. I have been given the authority and autonomy to help solve customer needs | 4 | 3 | 2 | 1 |

"Emotional demands and regulation" 1-5
"Overload and conflict in customer service" 6-8
"Emotional disharmony and hurt" 9-14
"Organizational surveillance and monitoring" 15-17
"Organizational support and protective systems" 18-24

| The following questionnaire was created to assess the level of experience of customer violence and workplace violence. Based on the business performance of the past year, please respond to the following questionnaire by selecting the option that most closely reflects your thoughts. |
| --- |

| Question | None | Sometimes | Often | Very often |
| --- | --- | --- | --- | --- |
| 1. I have heard bad things such as unpleasant accusations or people shouting while conducting business | 1 | 2 | 3 | 4 |
| 2. I have faced sexual harassment while conducting business | 1 | 2 | 3 | 4 |
| s3. I have been threatened or bullied by customers while conducting business | 1 | 2 | 3 | 4 |
| 4. Sometimes customers face discrimination regarding job title, gender, and age during the course of business | 1 | 2 | 3 | 4 |
| 5. I have heard bad things at the workplace such as insults, accusations, or people shouting at the boss and my colleagues | 1 | 2 | 3 | 4 |
| 6. Sometimes I have to deal with unnecessary sexual or physical contact and harassment by my boss and colleagues at my workplace | 1 | 2 | 3 | 4 |
| 7. I have received threats and been harassed and bullied by my boss and colleagues at my workplace | 1 | 2 | 3 | 4 |
| 8. I have faced discrimination regarding job title, gender, or age by my boss and colleagues at my workplace | 1 | 2 | 3 | 4 |
| 9. While conducting business, I may  have been physically assaulted by a customer | 1 | 2 | 3 | 4 |
| 10. I have been physically assaulted by my boss and colleagues at my workplace | 1 | 2 | 3 | 4 |

| The following questionnaire was created to evaluate how workplace protection and management of customer and workplace violence are being conducted. Based on the business performance of the past year, please respond to the following questionnaire by selecting the option that most closely reflects your thoughts. | | | | |  |
| --- | --- | --- | --- | --- | --- |
| Question | Strongly disagree | Disgree | Agree | Strongly agree | |
| 11. Devices and institutions are established to prevent violence in the workplace | 4 | 3 | 2 | 1 | |
| 12. The workplace implements various measures so as not to match violence to customers . | 4 | 3 | 2 | 1 | |
| 13. There are educational programs and action guidelines that can deal with customer violence in the workplace | 4 | 3 | 2 | 1 | |
| 14. The workplace protects me so that I can work safely without experiencing violence | 4 | 3 | 2 | 1 | |
| 15. In the event of a problem caused by customer violence in the workplace, there are internal regulations | 4 | 3 | 2 | 1 | |
| 16. Equipment and systems to prevent violence from colleagues and superiors in the workplace have been established | 4 | 3 | 2 | 1 | |
| 17. Various measures have been taken in my workplace does not match violence from colleagues or boss | 4 | 3 | 2 | 1 | |
| 18. Guidelines are established through educational programs that deal with the violence of colleagues and bosses in the workplace | 4 | 3 | 2 | 1 | |
| 19. I feel safe and protected in my workplace and do not experience violence from my colleagues and boss | 4 | 3 | 2 | 1 | |
| 20. In the event of violence from colleagues or bosses in the workplace, there are internal regulation s | 4 | 3 | 2 | 1 | |
| 21. There are supervisors who will help solve problems when one  gets violent | 4 | 3 | 2 | 1 | |
| 22. There are companies that will help solve problems when one gets violent. | 4 | 3 | 2 | 1 | |
| 23. The workplace empathizes with those who have been emotionally scarred from physical assault and tries to rectify these wounds | 4 | 3 | 2 | 1 | |
| 24. The workplace solves problems that occur during the process of violence | 4 | 3 | 2 | 1 | |

"experience  of psychological and sexual violence from customer" 1-4
"experience of psychological and sexual violence from supervisor and coworker" 5-8
"experience of physical violence from customer, supervisor and coworker" 9-10
"organizational protective system for workplace violence" 11-24
